# Diagnostic value of neutrophil‐to‐lymphocyte and platelet‐to‐lymphocyte ratio to predict recurrent pregnancy loss and abortion; a systematic review and meta‐analysis

**DOI:** 10.1002/iid3.1210

**Published:** 2024-03-20

**Authors:** Sedigheh Hantoushzadeh, Omid Kohandel Gargar, Kyana Jafarabady, Mohammad Moein Rezaei, Fatemeh Asadi, Nasim Eshraghi, Zahra Panahi, Saeedeh Shirdel, Masoumeh Mirzamoradi, Marjan Ghaemi

**Affiliations:** ^1^ Vali‐E‐Asr Reproductive Health Research Center, Family Health Research Institute Tehran University of Medical Sciences Tehran Iran; ^2^ Student Research Committee Alborz University of Medical Sciences Karaj Iran; ^3^ Clinical Research Development Center, Mahdiyeh Educational Hospital Shahid Beheshti University of Medical Sciences Tehran Iran

**Keywords:** inflammatory markers, missed abortion, recurrent pregnancy loss, systematic review, threatened abortion

## Abstract

**Objective:**

This systematic review and meta‐analysis aimed to evaluate the diagnostic value of the neutrophil‐to‐lymphocyte ratio (NLR) and platelet‐to‐lymphocyte ratio (PLR) in women with a history of abortion (missed and threatened) and recurrent pregnancy loss (RPL) in comparison with healthy pregnancies.

**Methods:**

Electronic databases including MEDLINE, Scopus, Web of Science, Embase, and Cochrane Library were searched for NLR and PLR in women who experienced early pregnancy loss up to January 1, 2023 with a combination of proper keywords. Meta‐analysis was done for comparison with three or more studies and summary estimates were measured.

**Results:**

A total of 390 citations were retrieved initially, and after screening, 16 articles were deemed eligible for the final review. Among these, 14 studies underwent meta‐analysis. The meta‐analysis revealed that the standard mean of the NLR was significantly higher in abortion cases compared to the control group. However, there was no significant difference in the PLR between the pregnancy loss group and the control group.

**Conclusion:**

NLR was significantly higher among RPL patients compared to the control group, according to these data, NLR may be capable of being used in the diagnosis of RPL as an easy, cheap, and accessible modality. Further studies, which take these variables into account, will need to be undertaken to determine the diagnostic value of NLR and PLR in early pregnancy loss.

## Graphical abstract

In a review of 16 eligible articles, meta‐analysis showed that the standard mean of the neutrophil‐to‐lymphocyte ratio (NLR) was significantly higher in cases of abortion compared to the control group. However, there was no significant difference in the platelet‐to‐lymphocyte ratio (PLR) between the pregnancy loss group and the control group. These findings suggest that NLR may have potential as an accessible and cost‐effective diagnostic tool for recurrent pregnancy loss (RPL), but further studies considering additional variables are needed to determine the diagnostic value of NLR and PLR in early pregnancy loss.

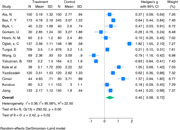

## 1 INTRODUCTION

Almost 10% of clinically recognized pregnancies result in early pregnancy loss.^1^ The majority of losses before 12 weeks of gestation are associated with fetal chromosomal abnormalities, which become more prevalent with advanced maternal age.^2^ Other causes included infections, immunological and endocrine disorders as well as anatomic factors, or genetics associated with pregnancy loss.^3^ However, the underlying reason for the majority of pregnancy losses is unknown.^2^ The 2021 rate of miscarriage was estimated at 23 million worldwide, and the population prevalence of women who have had three or more miscarriages was 0.7%.^4^ Miscarriage has plenty of psychological consequences including depression, anxiety, posttraumatic stress syndrome, and even suicide, needless to say, a miscarriage would cost lots of economic adverse due to the obstetrics and psychological complications which require future healthcare services.^4^

A regulated inflammatory environment for efficient implantation and tissue remodeling is mandatory in a normal pregnancy. Thus, the upregulation of cytokines is the main feature of this process.^5^ However, the majority of abortions have occurred because of problems with immunological mechanisms and inflammatory processes.^6^ Intravascular inflammatory reaction including leukocytes, platelets, and complement system is followed by endothelial dysfunction.^7^ When inflammation occurs, there is a rise in neutrophil and platelet count and a fall in lymphocyte count in blood circulation, which is the reason for the high neutrophil‐to‐lymphocyte ratio (NLR) ratio in inflammatory processes.^8^

Using inflammatory markers such as NLR and platelet‐to‐lymphocyte ratio (PLR), we can assess the presence and the load of the inflammatory process which can be used for finding possible treatments for recurrent pregnancy loss and abortion. These markers are easily obtained from a complete blood count (CBC) test.

This study aims to evaluate and validate:

1. Providing an overview of studies identifying the relationship between the NLR and PLR values and recurrent pregnancy loss and abortion.
2. Assessing the prognostic value of each one of the markers in predicting abortion.
3. Determine which marker is more useful in the diagnosis/prognosis of recurrent pregnancy loss and abortions.

## 2 METHOD

The current systematic review was designed to review the NLR and PLR relation with early pregnancy loss in English biomedical journals. The study is reported based on the Preferred Reporting Items for Systematic Reviews and Meta‐Analyses (PRISMA) statement.^9^

### 2.1 Eligibility criteria

The inclusion criteria were the observational studies that compared NLR and PLR in patients with either spontaneous miscarriage, recurrent pregnancy loss or threatened abortion to healthy controls and met the following inclusion criteria were found eligible:

1) Study types: In any type of published controlled observational study, the language is limited to English.
2) Study subjects: For patients with early pregnancy loss, the diagnostic criteria according to the American College of Obstetricians and Gynecologists (ACOG), it is characterized as a nonviable, intrauterine gestation with either an empty gestational sac or a gestational sac containing a fetus without embryonic cardiac activity in ultrasound within the first 12 6/7 weeks of gestation.^1^ RPL was defined as three or more pregnancy losses that occur before 24 gestation weeks.^10^ Threatened abortion is defined as vaginal bleeding before 20 weeks gestation without dilated cervical os.^11^ Missed abortion means an empty gestational sac or lack of fetal heart rate before 20 weeks of gestational age.
3) Study reports: the prognostic impact of the blood NLR and PLR, collected before the event, reporting the hazard ratios and corresponding 95% CI and/or *p* value.

Exclusion criteria were non‐spontaneous pregnancy loss, studies without comparison groups (case reports, case series, etc.), review articles, opinion pieces or guidelines, non‐peer‐reviewed papers, unpublished reports, and articles in which the date and location of the study were not specified. Studies showing data in graphic form without documenting a numerical value for hazard ratios were also excluded.

### 2.2 Information sources

We performed a computerized search of published relevant studies from the database of PubMed, Web of Science, Scopus, Embase, and Cochrane Library, for studies conducted from inception to January 1, 2023.

### 2.3 Search strategy

The search started with a combination of proper terms that is summarized in Supporting Information File 1. We aimed to pinpoint all relevant studies, regardless of the type of publication. A combination of subject headings and free words from the database establishment were used.

### 2.4 Study selection

After duplicate removal, K.J. and M.R. independently screened titles and abstracts and excluded nonrelevant studies and studies that did not meet the inclusion criteria. The remaining went through full‐text screening and eligibility assessment.

### 2.5 Data extraction

Two expert physicians (K.J. and M.R.) investigated the articles independently and extracted the data according to the predetermined screening criteria. A third party was involved to make the extracted data unanimous. The articles that were retrieved electronically were screened by two expert physicians. The initial screening was accomplished by reading the title and abstract of the studies, those who did not meet the inclusion criteria were excluded in this step. The full text of potentially eligible studies was meticulously read and re‐screened before being included. The authors of the articles were contacted by e‐mail if any important data was missing or ambiguous. Data extracted from the included studies comprised: (1) the first author, (2) publication year, (3) sample size, (4) median age, (5) collection of data (retrospective, prospective), (6) gestational age, (7) cut off value used to define high NLR and PLR, (8) receiver operating characteristic curve analysis, and (9) hazard ratios and associated 95% CIs.

### 2.6 Risk of bias assessment

The validity of studies was evaluated by two investigators using the National Heart, Lsung, and Blood Institute risk of a bias assessment tool for case–control studies.^12^ Studies scoring 9 or more were marked as “Good,” studies scoring between 7 or 8 were marked as “Fair” and studies rating less than 7 were marked as “Poor.”

### 2.7 Outcomes

There are only two outcomes in this study, NLR and PLR.

### 2.8 Registration statement

The protocol of this study was registered and approved by the ethics review board of the Tehran University of Medical Sciences.

### 2.9 Synthesis method

Due to the heterogeneous nature of NLR and PLR, the random effect model was used for data synthesis. In each comparison pooled standard mean difference with corresponding 95% confidence interval (CI) of NLR and PLR is measured using Hedges g. Comparisons with less than three studies are not pooled and summary estimates are not measured, findings of these studies are reported without overall estimates measured. Heterogeneity among studies was measured using Cochran's *Q* test and the quared statistic. A method proposed by Wan et al. was employed to convert median and interquartile range (IQR) to mean and standard deviation in studies that reported their outcomes in median and IQR.^13^ To assess possible bias caused by using this method a sub‐group analysis was performed and the significance of the subgroup differences was measured. To investigate publication bias, funnel plots were constructed and assessed using Egger's and Begg's tests. All statistical analyses were performed using Stata 17.0 software (Stata Corp). A two‐tailed *p* < .05 was considered significant.

### 2.10 Ethical consideration

There was no contact with humans in this study, so ethical approval was waived by the Tehran University of Medical Sciences Ethics Committee.

## 3 RESULTS

### 3.1 Included studies

**Figure 1:**
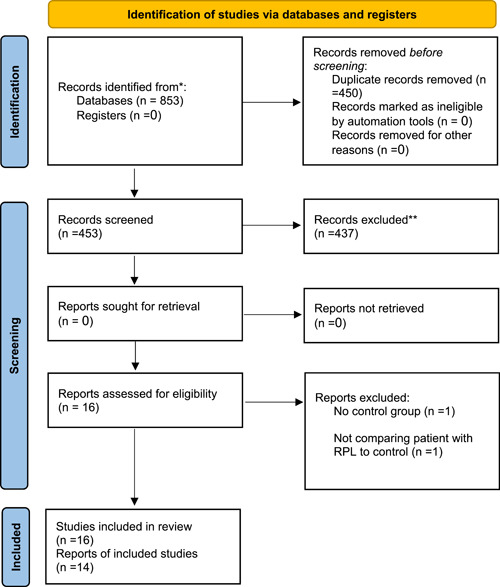
Preferred reporting items for systematic reviews and meta‐analyses (PRISMA) flowchart. PRISMA 2020 flow diagram for new systematic reviews, which included searches of databases and registers only.

The systematic search resulted in the identification of 853 potentially relevant articles. After a thorough review, 16 studies were included in the analysis. Finally, based on the predefined inclusion and exclusion criteria, a total of 14 studies were selected for the final meta‐analysis. The PRISMA flowchart (Figure 1) illustrates the selection process.

Table 1 summarizes the main characteristics of the included studies. These studies were conducted in various countries, with sample sizes ranging from 150 to 2500 participants. They encompassed a diverse range of populations and settings, with data collected over the past two decades.

**Table 1:** Characteristics of included studies quality assessment.

1st author	Year	Country	Groups		Number	Time of evaluation (gestational week)	Quality
Ata, N^14^	2020	Turkey	Threatened abortion		100	7–14th weeks	Fair
			Missed abortion		100		
			Control		100		
Bas, F. Y^15^	2018	Turkey	Missed abortion	First trimester	173	Before 6th week	Fair
				Second trimester	152		
			Control		245		
Biyik, I.^16^	2020	Turkey	Missed abortion		40	Before 13th weeks	Fair
			Control		40		
Christoforaki, V.^17^	2020	Greece	Missed abortion		64	7–14th weeks	Fair
			Control		65		
Cimsir, M. T.^18^	2021	Turkey	Recurrent pregnancy loss		44	Immediately after the diagnosis	Good
			Control		60		
Gorkem, U.^19^	2021	Turkey	Threatened abortion		30	7–9th weeks	Fair
			Missed abortion		30		
			Control		30		
Hosni, N. M.^20^	2021	Egypt	Control		60	6–13th weeks	Fair
			Missed abortion		60		
			Ectopic pregnancy		30		
Jiang, S.^21^	2021	China	Recurrent pregnancy loss		133	At least 12 weeks after the last abortion	Good
			Control		140		
Liu, D^22^	2022	China	Missed abortion		200	Before 13th weeks	Fair
			Control		200		
Oğlak, S. C.^23^	2020	Turkey	Missed abortion		137	In the early pregnancy loss group CBC was determined at the time of referral to the hospital for routine follow‐up, In the control group, before the seventh week	Fair
			Control		148		
Turgut, E.^24^	2022	Turkey	Control		676 709	N.R	Fair
			Missed abortion				
Wang, Q^25^	2020	China	control		53	7–13th weeks	Fair
			Missed abortion	Without progesterone treatment	53		
				With progesterone treatment	16		
Yakıştıran, B.^26^	2021	Turkey	Elective abortions		32	7th week	Fair
			Missed abortion		193		
			Control		164		
			Ectopic pregnancy		152		
			Missed abortion		80		
			Control		48		
Kale et al.^27^	2021	Turkey	Missed abortion		39	First trimester	Fair
			Control		200		
Karakus^28^	2016	Turkey	Control		153	First trimester	Fair
			Missed abortion		80		
Yazdizadeh^29^	2023	Iran	Control		120 2	6–13th weeks	Good
			Missed abortion		120		

#### 3.1.1 NLR

**Figure 2:**
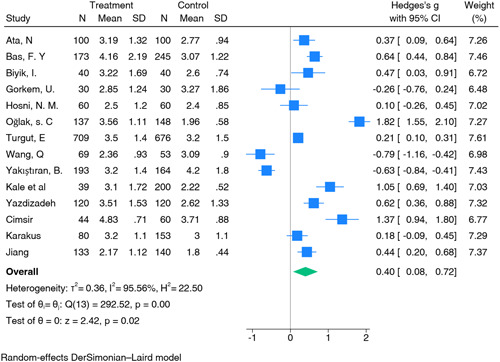
Neutrophil‐to‐lymphocyte ratio. CI, confidence interval.

**Figure 3:**
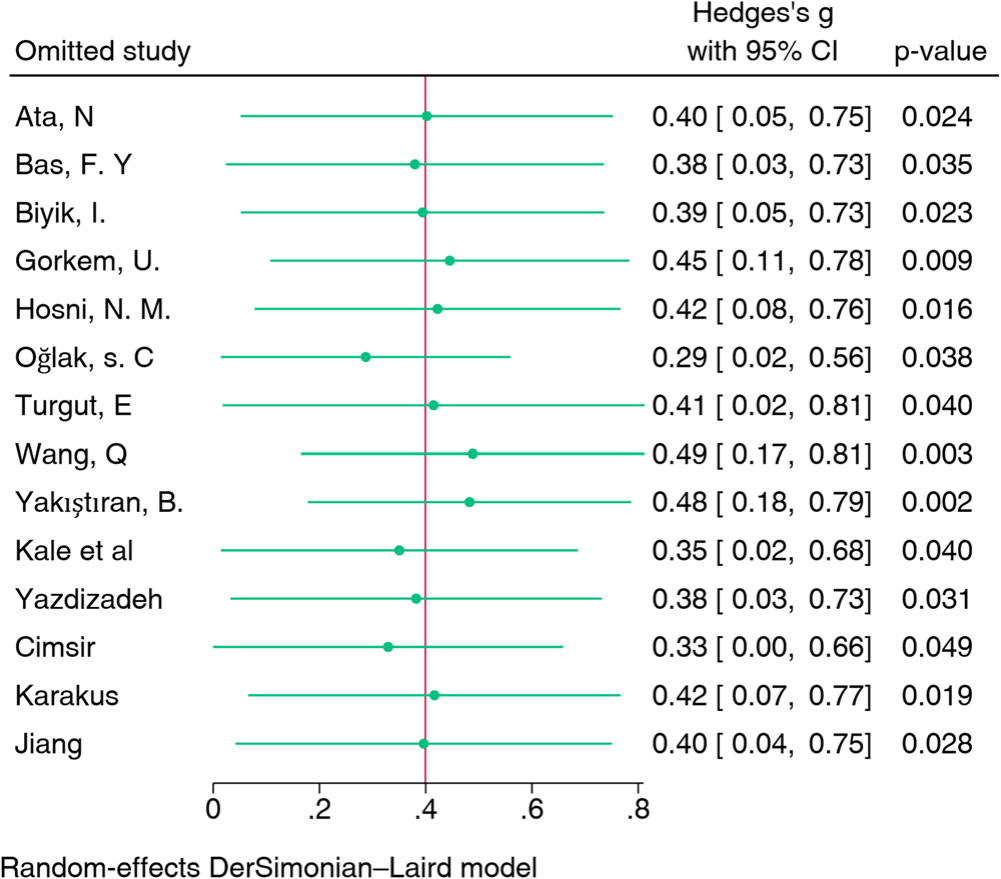
Sensitivity analysis of neutrophil‐to‐lymphocyte ratio. CI, confidence interval.

A comprehensive analysis was conducted on 14 studies examining the correlation between NLR and early pregnancy loss. These studies encompassed a total of 1927 cases of pregnancy loss and 2189 controls (Table 2). The results of the analysis demonstrated a statistically significant effect (*p* < .001). The summary estimate (SMD) was determined to be 0.40 (95% CI: 0.08–0.72), suggesting that an elevated NLR is linked to a higher risk of early pregnancy loss. High heterogeneity among studies was observed (*I*² = 95%, *p* < .01) (Figure 2). Sensitivity analysis, which involved the removal of individual studies to assess their impact on the overall effect, did not reveal any single study that significantly influenced the summary estimates for NLR (Figure 3).

**Table 2:** Characteristics of included studies for neutrophil‐to‐lymphocyte ratio.

Study	mean1	SD1	number1	mean2	SD2	number2	pop
Ata, N	3.19	1.32	100	2.77	0.94	100	Abortion
Bas, F. Y	4.16	2.19	173	3.07	1.22	245	Abortion
Biyik, I.	3.22	1.69	40	2.6	0.74	40	Abortion
Gorkem, U.	2.85	1.24	30	3.27	1.86	30	Abortion
Hosni, N. M.	2.5	1.2	60	2.4	0.85	60	Abortion
Oğlak, S. C	3.56	1.11	137	1.96	0.58	148	Abortion
Turgut, E	3.5	1.4	709	3.2	1.5	676	Abortion
Wang, Q	2.36	0.93	69	3.09	0.9	53	Abortion
Yakıştıran, B.	3.2	1.4	193	4.2	1.8	164	Abortion
Kale et al.	3.1	1.72	39	2.22	0.52	200	Abortion
Yazdizadeh	3.51	1.53	120	2.62	1.33	120	Abortion
Cimsir	4.83	0.71	44	3.71	0.88	60	RPL
Karakus	3.2	1.1	80	3	1.1	153	Abortion
Jiang	2.17	1.12	133	1.8	0.44	140	RPL

#### 3.1.2 PLR

**Figure 4:**
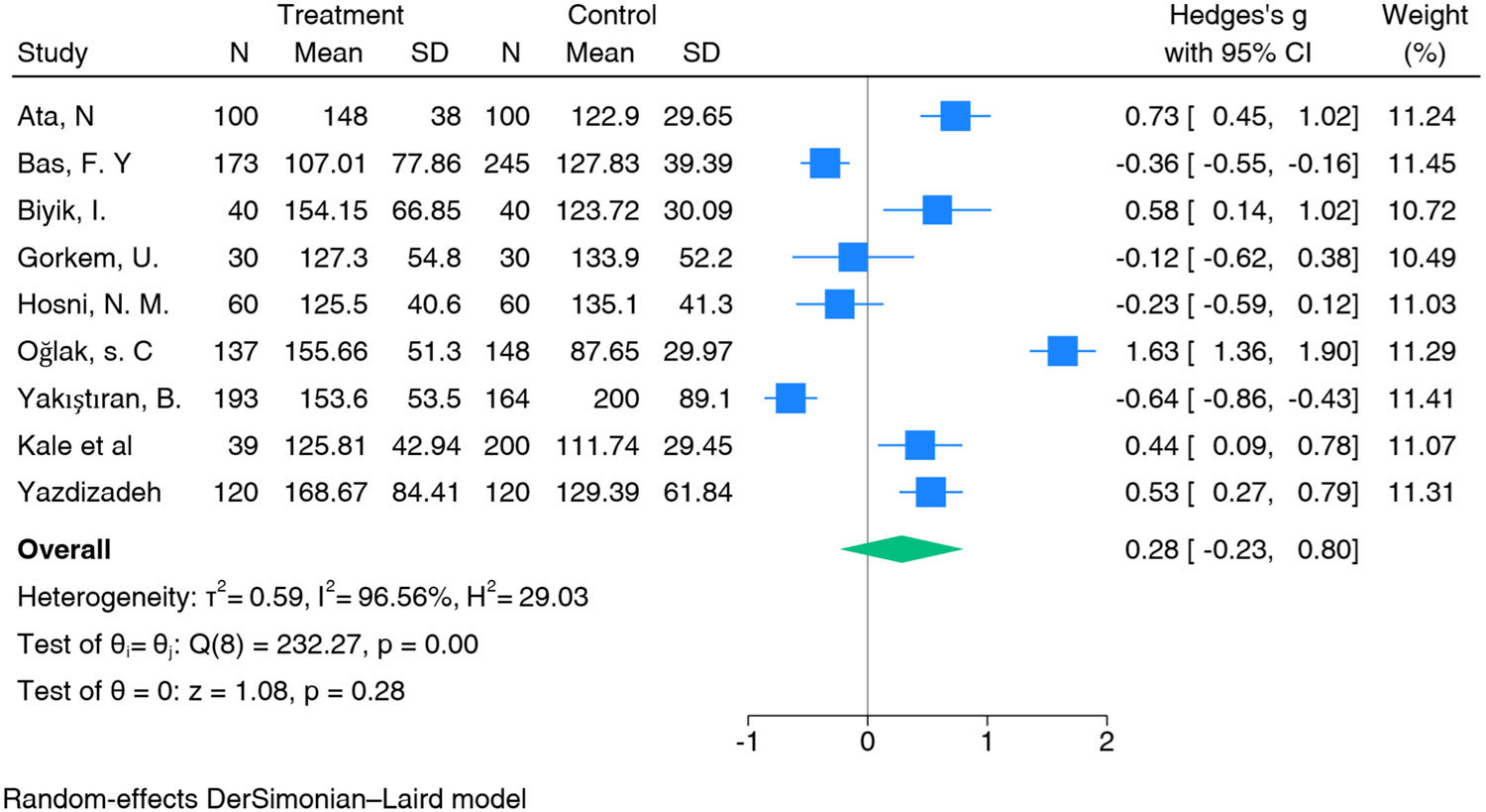
Platelet‐to‐lymphocyte ratio. CI, confidence interval.

**Figure 5:**
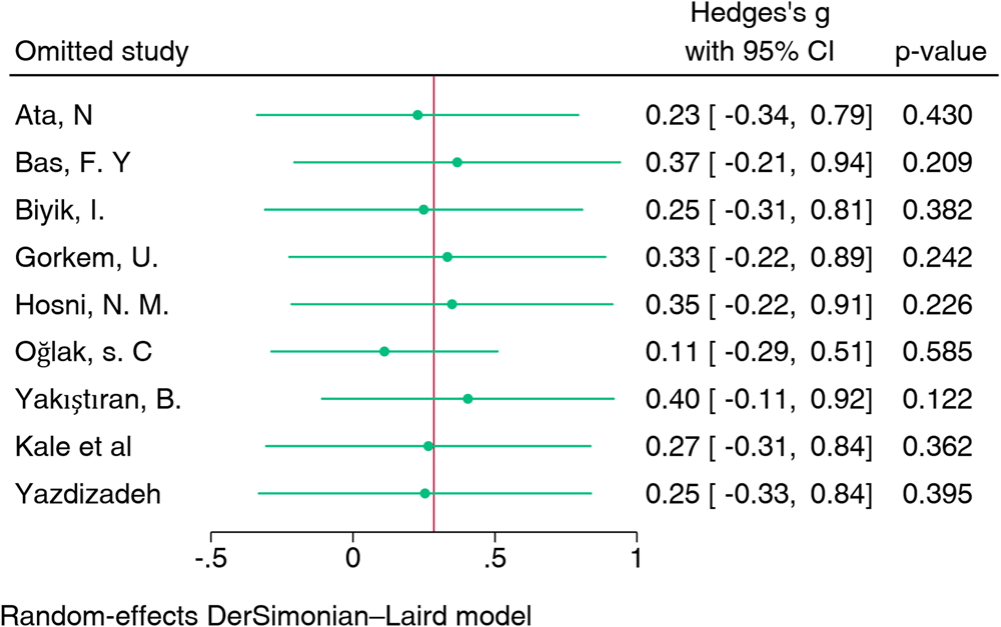
Sensitivity analysis of platelet‐to‐lymphocyte ratio. CI, confidence interval.

The meta‐analysis of 9 studies investigating the association between 892 PLR and 1107 early pregnancy loss (Table 3) did not show a statistically significant effect. The summary estimate was 0.28 (95% CI: −0.23‐0.80) (Figure 4). Heterogeneity was observed (I² = 96%, *p* < .01), suggesting high variability in study results. Sensitivity analysis, which involved the removal of individual studies to assess their impact on the overall effect, did not reveal any single study that significantly influenced the summary estimates for PLR (Figure 5).

**Table 3:** Characteristics of included studies for platelet‐to‐lymphocyte ratio.

Study	mean1	SD1	number1	mean2	SD2	number2
Ata, N	148	38	100	122.9	29.65	100
Bas, F. Y	107.01	77.86	173	127.83	39.39	245
Biyik, I.	154.15	66.85	40	123.72	30.09	40
Gorkem, U.	127.3	54.8	30	133.9	52.2	30
Hosni, N. M.	125.5	40.6	60	135.1	41.3	60
Oğlak, S. C	155.66	51.3	137	87.65	29.97	148
Yakıştıran, B.	153.6	53.5	193	200	89.1	164
Kale et al.	125.81	42.94	39	111.74	29.45	200
Yazdizadeh	168.67	84.41	120	129.39	61.84	120

#### 3.1.3 Publication bias

**Figure 6:**
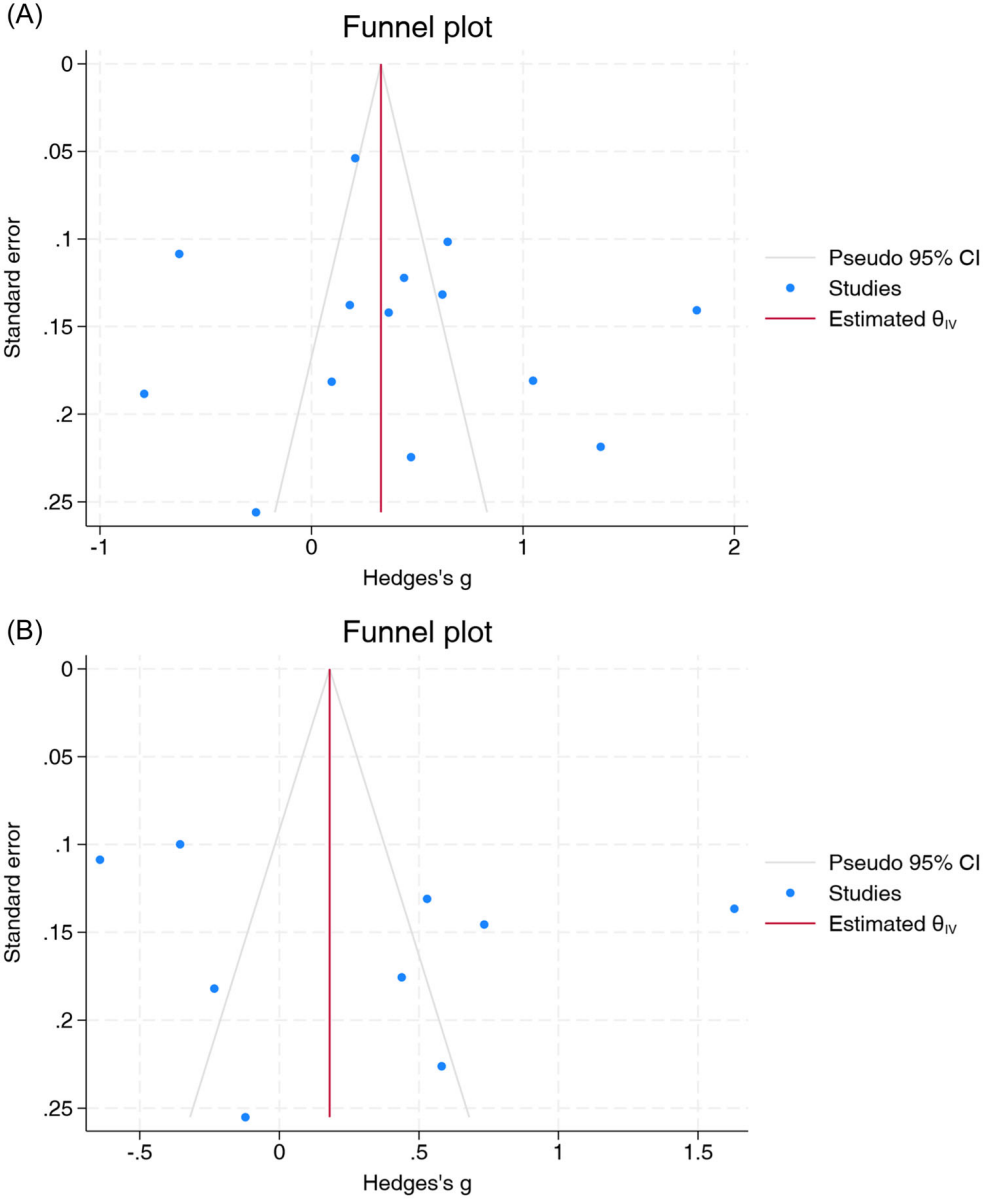
Funnel plots of (A) neutrophil‐to‐lymphocyte ratio and (B) platelet‐to‐lymphocyte ratio. CI, confidence interval.

Visual inspection of funnel plots and Egger's test did not indicate potential publication bias for both NLR and PLR (*p* > .05) (Figure 6).

#### 3.1.4 Quality of evidence

The overall quality of evidence for both NLR and PLR was rated as moderate, with the potential for bias and heterogeneity being the main limitations.

## 4 DISCUSSION

This is the first systematic review and meta‐analysis study about NLR and PLR in patients with abortion disorders. NLR and PLR are compared between patients group included (spontaneous abortion threatened abortion and recurrent pregnancy loss) and healthy controls. Our results revealed a significant increase in the amount of NLR among patients with early pregnancy loss compared to healthy control groups, although a high amount of heterogeneity was observed among studies. PLR has not shown a significant difference between these two groups. The sensitivity analysis did not identify any particular study that significantly affected the summarized estimates for NLR and PLR.

Up to 50% of RPL cases do not have a clear etiology but multifactorial conditions such as immunological, anatomical, genetic, and hematological disorders are known to be the causes of RPL. Inflammation and stress play an important role in the process of unsuccessful implantation and pregnancy.^30^ In this connection, inflammatory factors such as neutrophils and lymphocytes may play an essential role in the pathophysiology of pregnancy loss.

In all studies, blood sampling was conducted during the first trimester, but the timing varied. The choice of timing may be attributed to the reported initiation of inflammation response from the implantation phase.^31^ Another study also reported that the earliest gestational age showing an inflammatory response was 4 weeks gestation.^32^

In our research, we included studies that examined NLR and PLR during pregnancy, acknowledging the variability in the timing of blood drawn across these studies. It is important to note that various inflammatory events, such as placental histiocytosis, can begin before pregnancy and potentially lead to miscarriage. For patients with RPL, interventions such as intravenous immunoglobulin and progesterone have shown potential efficacy in addressing these inflammatory pathways.^33^, ^34^ However, our study focused on determining the predictive role of NLR and PLR during pregnancy in individuals experiencing RPL.

NLR is a known value that has been reported to be related to cancer growth, metastasis, poor response to treatment, and worse survival.^35^ In the study by Bonavita et al.,^36^ rise in the number of neutrophils inhibits the immune system by suppressing the activity of lymphocyte and Tcell response. NLR also can have a significant role in pregnancy complications such as pre‐eclampsia; patients who have been diagnosed with pre‐eclampsia have higher amounts of NLR and lower PLR^37^ and also NLR is reported to be a predictor for the severity of pre‐eclampsia.^38^ Another pregnancy complication that is related to higher NLR is intrahepatic cholestasis of pregnancy and also NLR can be a predictor of severity in this complication too.^39^

Normal pregnancy is known as a hypercoagulable state and needs sterile inflammation for successful implantation;^40^ however, if uncontrolled implantation occurs, it may lead to deteriorated maternal health,^41^ which causes an increase in the number of NK cells and secretion of cytokines that provides an ischemic situation for embryos by activation of vascular endothelial cell procoagulants that results in an inflammatory and coagulation condition^42^, ^43^ that can lead to an increased number of neutrophils that as it mentioned suppress the lymphocytes and results in higher NLR and also coagulation stats that happen during pregnancy loss can be a reason for increasing platelet.

Several significant limitations need to be considered; the most important limitation lies in the fact that the number of studies that have conducted the association between NLR, PLR value, and RPL, threatened and missed abortion, were small. Another limitation that has resulted in the exclusion of some studies was the comorbidities, which could lead to solid bias; a good illustration is PLR value in women who are diagnosed with thrombophilia and pregnancy loss.

It is important to note that in our study, each patient with RPL had previously experienced a single miscarriage. For future studies, it is recommended to investigate the role of NLR and PLR in individuals facing threatened abortion. Additionally, exploring the potential benefits of progesterone treatment in suppressing the inflammatory processes associated with threatened abortion would be an important area for further research.

To the best of our knowledge, our study is the first to review the diagnostic value of NLR and PLR in RPL and abortions. Further studies, which take these variables into account, will need to be undertaken. Clinical trials with a more significant number of participants needed to be done to compare the value of NLR and PLR between women who have experienced abortion or RPL and healthy pregnancies.

## 5 CONCLUSION

In conclusion, the available evidence suggests a significant association between early pregnancy loss and a higher NLR. As a result, we considered elevated NLR as a potential cause of miscarriage. However, it should be noted that there was considerable heterogeneity among the included studies. Sensitivity analysis revealed that the overall estimate's significance remained unchanged even with the inclusion of certain studies. On the other hand, the PLR did not show a significant difference between the patient groups and controls.

To further investigate the role of NLR and PLR in these pregnancy complications, additional studies with larger sample sizes and standardized methodologies are needed. Conducting more research in this area will provide a more comprehensive understanding of the significance of these ratios in the context of early pregnancy loss.

## AUTHOR CONTRIBUTIONS

**Sedigheh Hantoushzadeh**: Conceptualization; supervision. **Omid Kohandel Gargar**: Methodology; validation; writing—original draft. **Kyana Jafarabady**: Methodology; validation; writing—original draft. **Mohammad Moein Rezaei**: Formal analysis; methodology; writing—original draft. **Fatemeh Asadi**: Investigation; software. **Nasim Eshraghi**: Data curation; formal analysis; writing—review and editing. **Zahra Panahi**: Formal analysis; supervision; validation. **Saeedeh Shirdel**: Formal analysis; writing—review and editing. **Masoumeh Mirzamoradi**: Data curation; visualization. **Marjan Ghaemi**: Conceptualization; supervision.

## CONFLICT OF INTEREST STATEMENT

The authors declare no conflicts of interest.

## Supporting information

Supporting information.

## Data Availability

Data sharing is available by contacting the corresponding author. All data are available in manuscript and supplementary material.
