# Supplementary material for: Diagnostic value of neutrophil‐to‐lymphocyte and platelet‐to‐lymphocyte ratio to predict recurrent pregnancy loss and abortion; a systematic review and meta‐analysis
Source: Immun Inflamm Dis. 2024 Mar 20;12(3):e1210. doi: 10.1002/iid3.1210 (PMC10953208; doi:10.1002/iid3.1210)

# Supplementary Material

**The effect of Myo-inositol therapy on sperm quality and its biochemical factors: a systematic review and meta-analysis.**

**Supplementary Table 1-** PRISMA 2020 checklist

**Supplementary Table 2-** Search strategies for online databases

**Supplementary Table 3-** Characteristics of the included studies.

**Supplementary Figure 1-** Risk of bias assessment for each included study

**Supplementary Figure 2-** Funnel plot for total sperm motility

**Supplementary Figure 3-** Funnel plot for progressive sperm motility

This supplemental material has been provided by the authors to give readers additional information about their work.

**Supplementary Table 1.** PRISMA 2020 checklist

| **Section and Topic** | **Item #** | **Checklist item** | **Reported on page #** |
| --- | --- | --- | --- |
| **TITLE** | | |  |
| Title | 1 | Identify the report as a systematic review. | 1 |
| **ABSTRACT** | | |  |
| Abstract | 2 | See the PRISMA 2020 for Abstracts checklist. | 3, 4 |
| **INTRODUCTION** | | |  |
| Rationale | 3 | Describe the rationale for the review in the context of existing knowledge. | 5 |
| Objectives | 4 | Provide an explicit statement of the objective(s) or question(s) the review addresses. | 6 |
| **METHODS** | | |  |
| Eligibility criteria | 5 | Specify the inclusion and exclusion criteria for the review and how studies were grouped for the syntheses. | 6, 7 |
| Information sources | 6 | Specify all databases, registers, websites, organisations, reference lists and other sources searched or consulted to identify studies. Specify the date when each source was last searched or consulted. | 6 |
| Search strategy | 7 | Present the full search strategies for all databases, registers and websites, including any filters and limits used. | Suppl. |
| Selection process | 8 | Specify the methods used to decide whether a study met the inclusion criteria of the review, including how many reviewers screened each record and each report retrieved, whether they worked independently, and if applicable, details of automation tools used in the process. | 7 |
| Data collection process | 9 | Specify the methods used to collect data from reports, including how many reviewers collected data from each report, whether they worked independently, any processes for obtaining or confirming data from study investigators, and if applicable, details of automation tools used in the process. | 7, 8 |
| Data items | 10a | List and define all outcomes for which data were sought. Specify whether all results that were compatible with each outcome domain in each study were sought (e.g. for all measures, time points, analyses), and if not, the methods used to decide which results to collect. | 7 |
|  | 10b | List and define all other variables for which data were sought (e.g. participant and intervention characteristics, funding sources). Describe any assumptions made about any missing or unclear information. | 7 |
| Study risk of bias assessment | 11 | Specify the methods used to assess risk of bias in the included studies, including details of the tool(s) used, how many reviewers assessed each study and whether they worked independently, and if applicable, details of automation tools used in the process. | 8 |
| Effect measures | 12 | Specify for each outcome the effect measure(s) (e.g. risk ratio, mean difference) used in the synthesis or presentation of results. | 8 |
| Synthesis methods | 13a | Describe the processes used to decide which studies were eligible for each synthesis (e.g. tabulating the study intervention characteristics and comparing against the planned groups for each synthesis (item #5)). | 8 |
|  | 13b | Describe any methods required to prepare the data for presentation or synthesis, such as handling of missing summary statistics, or data conversions. | 8 |
|  | 13c | Describe any methods used to tabulate or visually display results of individual studies and syntheses. | 8 |
|  | 13d | Describe any methods used to synthesize results and provide a rationale for the choice(s). If meta-analysis was performed, describe the model(s), method(s) to identify the presence and extent of statistical heterogeneity, and software package(s) used. | 8 |
|  | 13e | Describe any methods used to explore possible causes of heterogeneity among study results (e.g. subgroup analysis, meta-regression). | 9 |
|  | 13f | Describe any sensitivity analyses conducted to assess robustness of the synthesized results. | 9 |
| Reporting bias assessment | 14 | Describe any methods used to assess risk of bias due to missing results in a synthesis (arising from reporting biases). | 8 |
| Certainty assessment | 15 | Describe any methods used to assess certainty (or confidence) in the body of evidence for an outcome. | 9 |
| **RESULTS** | | |  |
| Study selection | 16a | Describe the results of the search and selection process, from the number of records identified in the search to the number of studies included in the review, ideally using a flow diagram. | 10, Figure 1 |
|  | 16b | Cite studies that might appear to meet the inclusion criteria, but which were excluded, and explain why they were excluded. | Figure 1 |
| Study characteristics | 17 | Cite each included study and present its characteristics. | 10, suppl. |
| Risk of bias in studies | 18 | Present assessments of risk of bias for each included study. | 10, suppl. |
| Results of individual studies | 19 | For all outcomes, present, for each study: (a) summary statistics for each group (where appropriate) and (b) an effect estimate and its precision (e.g. confidence/credible interval), ideally using structured tables or plots. | Figures, suppl. |
| Results of syntheses | 20a | For each synthesis, briefly summarise the characteristics and risk of bias among contributing studies. | 10-12 |
|  | 20b | Present results of all statistical syntheses conducted. If meta-analysis was done, present for each the summary estimate and its precision (e.g. confidence/credible interval) and measures of statistical heterogeneity. If comparing groups, describe the direction of the effect. | 10-12 |
|  | 20c | Present results of all investigations of possible causes of heterogeneity among study results. | 11, 12, Table 1 |
|  | 20d | Present results of all sensitivity analyses conducted to assess the robustness of the synthesized results. | 11 |
| Reporting biases | 21 | Present assessments of risk of bias due to missing results (arising from reporting biases) for each synthesis assessed. | 11, 12 |
| Certainty of evidence | 22 | Present assessments of certainty (or confidence) in the body of evidence for each outcome assessed. | 11, 12, suppl. |
| **DISCUSSION** | | |  |
| Discussion | 23a | Provide a general interpretation of the results in the context of other evidence. | 12-14 |
|  | 23b | Discuss any limitations of the evidence included in the review. | 14 |
|  | 23c | Discuss any limitations of the review processes used. | 14 |
|  | 23d | Discuss implications of the results for practice, policy, and future research. | 14, 15 |

**Supplementary Table 2.** Search strategies for online databases

| **MEDLINE (via PubMed)** | 1.“Inositol” [mh] OR Inositol[tiab] OR Chiro-Inositol[tiab] OR Mesoinositol[tiab] OR Myoinositol[tiab]  2- “Semen” [mh] OR “Spermatozoa” [mh] OR Semen [tiab] OR Seminal [tiab] OR Male infertility[tiab] OR Male fertility[tiab] OR Male subfertility[tiab] OR Male sterility[tiab] OR Male reproductive system[tiab] OR Male reproduction[tiab] OR Male reproductivity[tiab] OR Sperm[tiab] OR Spermatozoa [tiab] OR Spermatozoon[tiab] OR Oligospermia[tiab] OR Cryptospermia[tiab] OR Cryptozoospermia[tiab] OR Hypospermatogenesis[tiab] OR Oligoasthenoteratozoospermia[tiab] OR Oligozoospermia[tiab] OR Azoospermia[tiab] OR Aspermia[tiab] OR total motile count[tiab] OR Progressive motility[tiab]  3- #1 AND #2 |
| --- | --- |
| **Embase** | 1- ‘Inositol’/exp OR ‘Inositol’:ti,ab OR ‘Chiro-Inositol’:ti,ab OR ‘Mesoinositol’:ti,ab OR ‘Myoinositol’:ti,ab  2- ‘Sperm’/exp OR ‘spermatozoon’/exp OR ‘Semen ‘:ti,ab OR ‘Seminal ‘:ti,ab OR ‘Male infertility’:ti,ab OR ‘Male fertility’:ti,ab OR ‘Male subfertility’:ti,ab OR ‘Male sterility’:ti,ab OR ‘Male reproductive system’:ti,ab OR ‘Male reproduction’:ti,ab OR ‘Male reproductivity’:ti,ab OR ‘Sperm’:ti,ab OR ‘Spermatozoa ‘:ti,ab OR ‘Spermatozoon’:ti,ab OR ‘Oligospermia’:ti,ab OR ‘Cryptospermia’:ti,ab OR ‘Cryptozoospermia’:ti,ab OR ‘Hypospermatogenesis’:ti,ab OR ‘Oligoasthenoteratozoospermia’:ti,ab OR ‘Oligozoospermia’:ti,ab OR ‘Azoospermia’:ti,ab OR ‘Aspermia’:ti,ab OR ‘total motile count’:ti,ab OR ‘Progressive motility’:ti,ab  3- #1 AND #2 |
| **Web of Science** | 1- “Inositol “OR “Chiro-Inositol” OR “Mesoinositol” OR “Myoinositol”  2- “Semen “ OR “Seminal “ OR “Male infertility” OR “Male fertility” OR “Male subfertility” OR “Male sterility” OR “Male reproductive system” OR “Male reproduction” OR “Male reproductivity” OR “Sperm” OR “Spermatozoa “ OR “Spermatozoon” OR “Oligospermia” OR “Cryptospermia” OR “Cryptozoospermia” OR “Hypospermatogenesis” OR “Oligoasthenoteratozoospermia” OR “Oligozoospermia” OR “Azoospermia” OR “Aspermia” OR “total motile count” OR “Progressive motility”  3- #1 AND #2 |

**Supplementary Table 3-** Characteristics of the included studies.

| **Author** | **Country** | **Type of study** | **Population** | **Total number of patients, n** | **Myoinositol (MI) theray details** | **Duration of treatment** | **Age, yrs** |
| --- | --- | --- | --- | --- | --- | --- | --- |
| Santoro et al., 2021 | Italy | prospective longitudinal study | Subjects aged over 18 years OAT patients Exclusion criteria: presence of varicocele, cryptorchidism, or prostatitis | 10 mixed samples from 30 OAT untreated patients (group A) then divided to the groups of B and C, each one 5 mixed samples from 15 OAT patients (treated in-vitro with MI and orally, respectively) | MI 2 mg/ml, 30min | 3 months | over 18 |
| Palmieri et al., 2016 | Italy |  | Men aged 22–60 years, including oligozoospermic, asthenozoospermic, and oligoasthenozoospermic subjects Exclusion criteria : cryptozoospermia, azoospermia, and ejaculate volume less than 1.5 mL. | 19 oligozoospermic subjects + 15 asthenozoospermic subjects + 20 oligoasthenozoospermic subjects + 25 thawed semen samples ( from patients aged 28–51 years) | 100 μl MI | NA | fresh :22–60/ thawed : 28–51 |
| Dinkova et al., 2017 | Bulgaria | prospective longitudinal study | 109 patients with asthenozoospermia | 109 | 1 g of MI, 30 mg of L-carnitine, L-arginine and Vitamin E, 55 μg of selenium, and 200 μg of folic acid , taken twice a day. | 3 months | 18 and 50 |
| Korosi et al., 2017 | Italy | prospective, randomized controlled trial | 22 couples undergo PICSI for male infertility issues inclusion criteria: age under 42 years OAT males and females with regular menstrual cycles (with hormonal profile: FSH (less than 10 mIU/ml), and Anti-Müllerian Hormone (AMH) (more than 1 ng/ml)) exclusion criteria: vasectomy, congenital absence of vas deferens, herniorrhaphy, hydrocelectomy, Young’s syndrome and ejaculatory duct obstruction | 22 couples | Case group (n=22): Oral treatment with 1 g of MI, 30 mg of L-carnitine, L-arginine and Vitamin E, 55 μg of selenium, and 200 μg of folic acid (Folandrol®, Exeltis, Hungary) twice a day [samples of the treated group = incubation for 2 h with 2 mg/ml of MI dispersed in the in vitro fertilization medium] Control group (n=13): No treatment in the same time | 2 months | under 42 |
| Saleh et al., 2017 | Egypt | A randomized controlled trial | infertile men | 25 | Each patient samples (1 mg) devided in 2 aliquots (0.5 ml each) for grop A and B:  Case group (A): treated with cryo-protectant plus 10 ml MI solution Control group (B): treated with cryo-protectant alone | between September 2016 and March 2017 | NA |
| De Leo et al., 2022 | Italy | prospective study | 36 Caucasian OAT patients Exclusion criteria: varicocele, cryptorchidism, endocrine disorders or systemic diseases and patients with intake of spermiotoxic drugs, smoking, alcohol or drugs abuse | 36  [Group A: 14 couple where male with supplementation before IVF Group B (control group): 14 couples where male with no supplementation before IVF] | Inositol (1000 mg) , L-Carnitine(250 mg ), Acetyl L-Carnitine Hydrochloride(250 mg) , Vitamin E(60 mg) , Vitamin C(100 mg) , Coenzyme Q10(20 mg) , Selenium(50 mcg) , Vitamin D3(5 mcg)/ Daily | 3 months | 25–47 years Male patient’s age(A):34.4 ± 6.8 Male patient’s age(B): 35.2 ± 6.3 |
| Montanino et al., 2016 | Italy | prospective longitudinal study | Asthenospermic males aged over 18 years with multiple sclerosis, under treatment at Altamedica IVF Unit Exclusion criteria: presence of cryptorchidism, varicocele, and prostatitis | 45 | 1g MI, 30mg L-carnitine, L-arginine and vitamin E, 55𝜇g selenium, and 200 𝜇g folic acid / twice a day | 3 months | over 18 years |
| Ghasemi et al., 2019 | Iran | double-blind randomized clinical trial | Men with oligoasthenospermia treated with intrauterine insemination (IUI) Inclusion criteria : the couples with male factor infertility (oligoasthenospermia or asthenospermia) during the last 12 months, despite the normal female factors, hysterosalpingography, and hormonal tests) Exclusion criteria: severe oligo-astheno-teratospermia (OAT) or other male factors, untreated hormonal problems, BMI > 35, genital abnormalities, and partner women aged over 38 yr | 37  (case group =13, control group = 24) | Case group= 0.5 ml of MI with a concentration of 2 mg/ml and incubated at 37°C for 2 hr Control group= no interventions, incubated for 2 hr at 37°C in 0.5 mL of the flushing medium in the special kit | 2 hr | men case group : 36.08 ± 5.88 control group : 33.96 ± 4.68 |
| Condorelli et al., 2012 | Italy |  | OAT Patients Exclusion criteria: a clinical history of cryptorchidism or varicocele, systemic or endocrine diseases, micro-orchidism, alcohol intake and/or drug abuse, male accessory gland infection, recent hormonal treatment, overweight/obese patients, and Smoking | 20 | MI (2 mg/mL) | 2 hr | 36.8 ± 3.2 |
| Artini et al., 2017 | Italy | in-vitro study | OAT patients Exclusion criteria: systemic and endocrine diseases, history positive for cryptorchidism or varicocele, male accessory gland infection, alcohol intake, recent hormonal treatment, and cigarette smoke and/or drug abuse | 32 | 15 ml/ml | 30 min | 35.5 ± 5.3 |
| Abdolsamadi et al., 2020 | Iran | in-vitro study | OAT patients referred to IVF center (with age between 28 and 45 years) | 40 | 2 ml/ml | 1 month | 28-45 |
| Azizi et al., 2022 | Iran | in-vitro study | Men (25–40 years old) with Asthenospermia (total motility <40%; progressive motility <32%) referred to the Infertility Center  Exclusion criteria: azoospermia, a history of cryptorchidism or varicocele, severe oligozoospermia, microrchidism, accessory gland infections, alcohol consumption, recent hormone treatment, and systemic diseases | 25 | 1, 2, 3 mg/ml Each semen sample was randomly divided into three groups: Fresh, MI (with freezing medium and 2mg/ml MI), and Control (with freezing medium alone) | NA | 25–40 years old |
| Capece et al., 2017 | Italy | single-centre, randomized, single-blind, placebo controlled study | OAT patients Exclusion criteria: systemic disorders, acute and chronic prostatitis, microorchidism and cryptorchidism, endocrine disorders, previous or current varicocele, previous penile or testicular infection, previous or current hormonal treatment, and smokers or ex-smokers | 60 | group A : MI 1000 mg, Tribulus Terrestris 300 mg, Alga Ecklonia Bicyclis 200 mg and Biovis, one tablet a day group B : One placebo tablet a day | 3 months | Group A : 28.4 ± 6.6 Group B : 28.8 ± 5.2 |
| Canepa et al., 2020 | Italy | clinical trial | No-smokers sub-fertile men (oligospermia and/or asthenospermia) aged 26-53 years (average age: 39.6 + 5.9 years), with one or more altered semen parameters according to WHO 2010 criteria | 100 | 2 tablets/day of Sinopol : contained ALA (800 mg), MI (1000 mg), folic acid (400 mg), betaine (100 mg), vitamin B2 (1.7 mg), B6 (1.9 mg), and B12 (2.6 mg) | 3 months | 37.7 + 4.7 years (28-52) |
| Calogero et al., 2015 | Italy | randomized, placebo-controlled double-blind study | patients younger than 45 years with idiopathic infertility  Exclusion criteria: identifiable cause of infertility (leukocytospermia and/or positive sperm culture, epididymo–orchitis, prostatitis, inguinoscrotal surgery, cryptorchidism, varicocele, etc.), azoospermia or severe oligozoospermia (sperm count less than 5 million/mL), | 194 | Group 1 (n = 98): Inofolic ( 2 g of MI and 200 lg of folic acid) twice daily Group 2 (n = 96): placebo(folic acid alone) twice daily | 3 months | group 1 : 28 ± 9 group 2 :28 ± 10 |
| Saleh et al., 2018 | Egypt | in-vitro study | semen samples refered to infertility center Exclusion criteria: leukocytospermia, azoospermia, or a volume less than 1 mL, history of antioxidants intake in the last 3 months prior to enrollment in the study | 26  [each semen sample was split into two equal aliquots (0.5 mL each) for MI and control group] | MI :0.5 mL of semen with 0.5 mL of sperm freezing medium + a dose of 10 μL/mL of MI CONTROL : 0.5 mL of semen with 0.5 mL of sperm freezing medium + 10 μL/mL of sperm washing and preparation medium | NA | 36 years [31–39] |

**Supplementary Figure 1.** Risk of bias assessment for each included study

**Supplementary Figure 2-** Funnel plot for total sperm motility


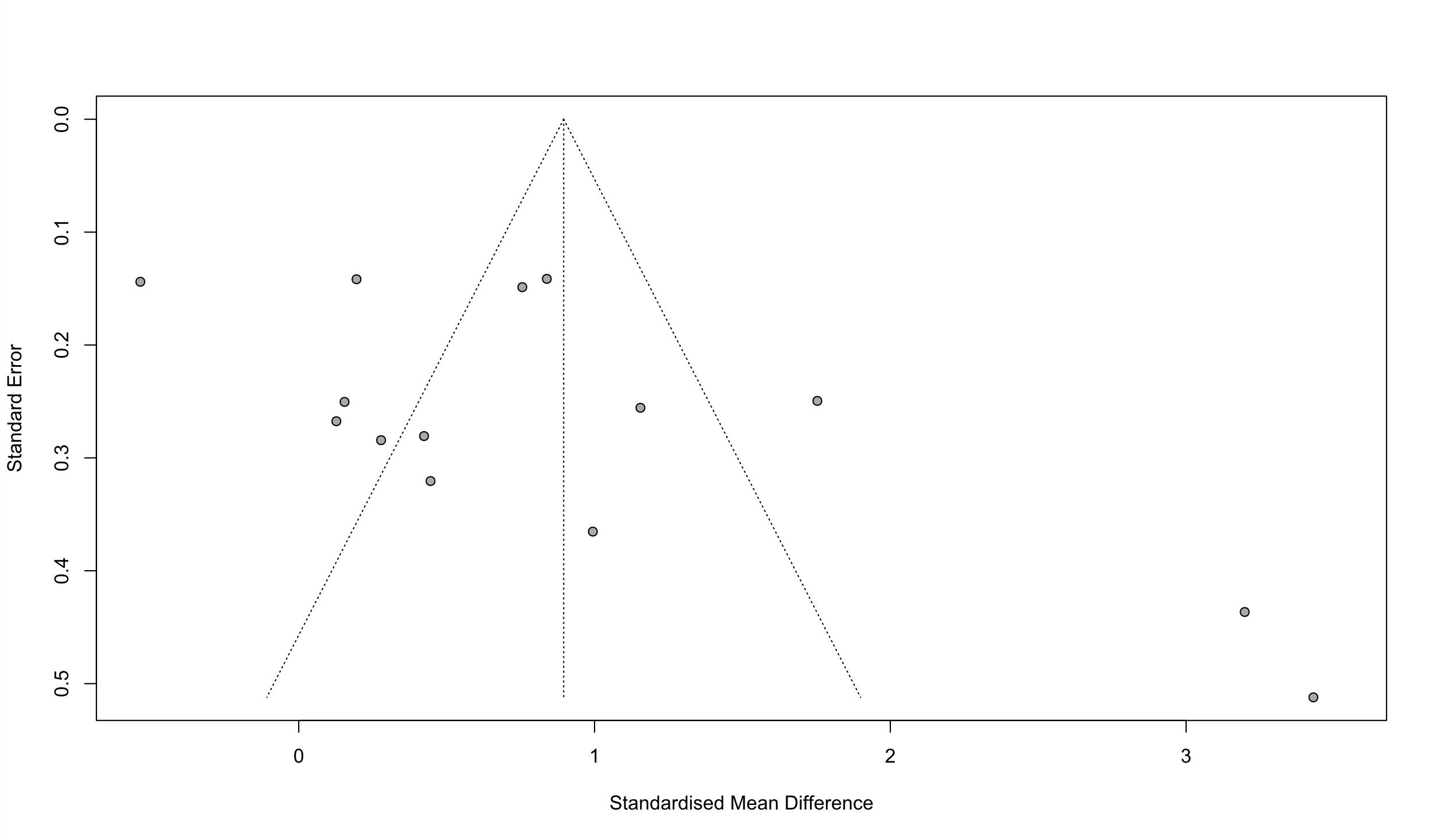


**Supplementary Figure 3-** Funnel plot for progressive sperm motility


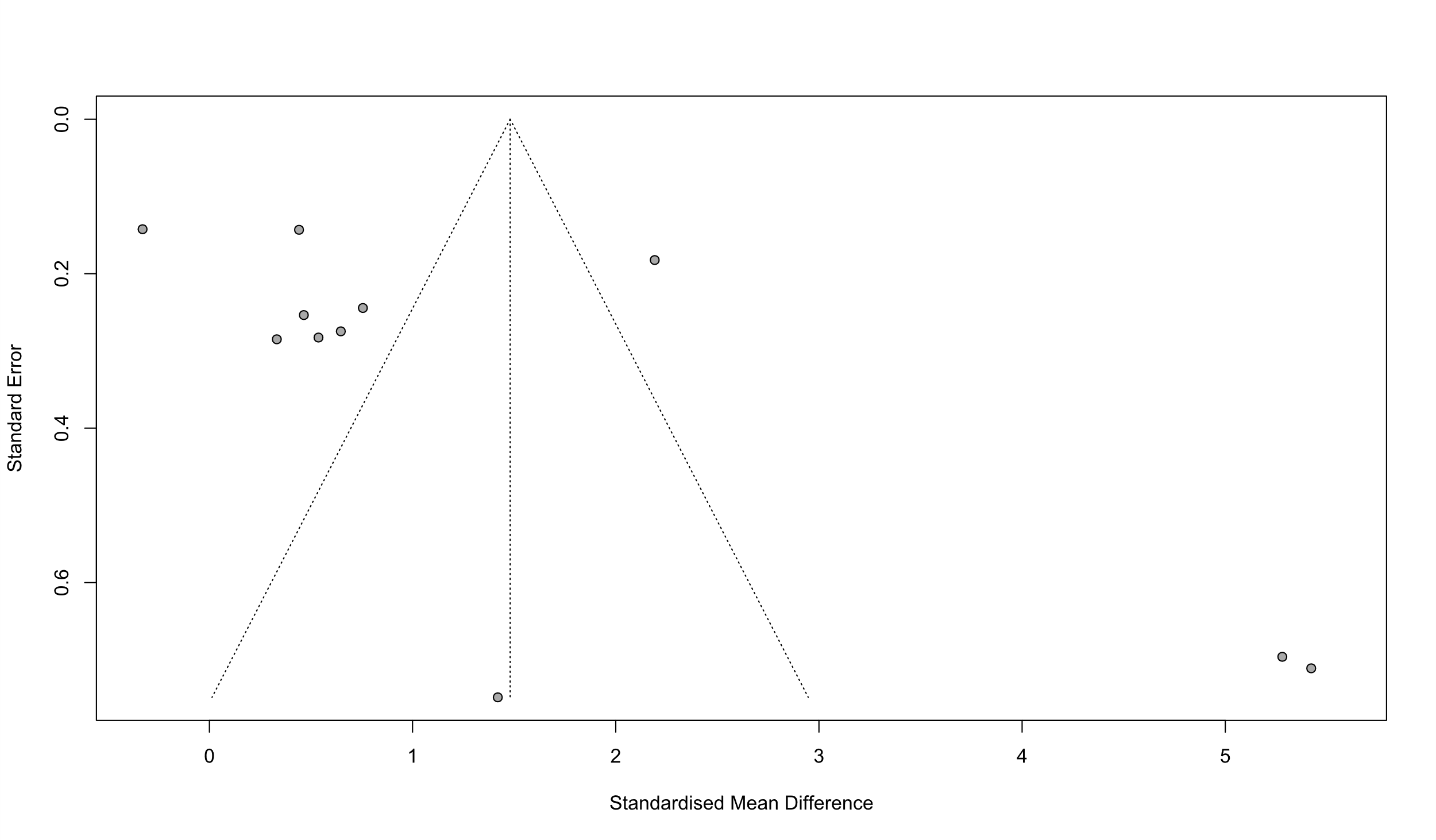

Supplement: Supplementary file 1 — Supporting information. [file IID3-12-e1210-s001.docx]
